# Identification and Mapping of HBsAg Loss-Related B-Cell Linear Epitopes in Chronic HBV Patients by Peptide Array

**DOI:** 10.3389/fimmu.2021.767000

**Published:** 2021-10-15

**Authors:** Shuqin Gu, Zhipeng Liu, Li Lin, Shihong Zhong, Yanchen Ma, Xiaoyi Li, Guofu Ye, Chunhua Wen, Yongyin Li, Libo Tang

**Affiliations:** ^1^ State Key Laboratory of Organ Failure Research, Guangdong Provincial Key Laboratory of Viral Hepatitis Research, Department of Infectious Diseases, Nanfang Hospital, Southern Medical University, Guangzhou, China; ^2^ Department of Oncology, Nanfang Hospital, Southern Medical University, Guangzhou, China

**Keywords:** B-cell linear epitopes, hepatitis B virus, HBsAg loss, peptide array, atypical memory B cells

## Abstract

Identification of immunogenic targets against hepatitis B virus (HBV)-encoded proteins will provide crucial advances in developing potential antibody therapies. In this study, 63 treatment-naïve patients with chronic HBV infection and 46 patients who achieved hepatitis B surface antigen loss (sAg loss) following antiviral treatment were recruited. Moreover, six patients who transitioned from the hepatitis B e antigen-positive chronic infection phase (eAg^+^CInf) to the hepatitis phase (eAg^+^CHep) were enrolled from real-life clinical practice. Additionally, telbivudine-treated eAg^+^CHep patients and relapsers or responders from an off-treatment cohort were longitudinally studied. The frequencies and function of B cells were assessed by flow cytometry. We devised a peptide array composed of 15-mer overlapping peptides of HBV-encoded surface (S), core (C), and polymerase (P) proteins and performed a screening on B-cell linear epitopes with sera. Naïve B cells and plasmablasts were increased, whereas total memory, activated memory (AM), and atypical memory (AtM) B cells were reduced in sAg^-^ patients compared with sAg^+^ patients. Importantly, longitudinal observations found that AtM B cells were associated with successful treatment withdrawal. Interestingly, we identified six S-specific dominant epitopes (S33, S34, S45, S76, S78, and S89) and one C-specific dominant epitope (C37) that reacted with the majority of sera from sAg^-^ patients. Of note, more B-cell linear epitopes were detected in CHep patients with alanine aminotransferase (ALT) flares than in nonflare CInf patients, and five B-cell linear epitopes (S4, S5, S10, S11, and S68) were overwhelmingly recognized by ALT flare patients. The recognition rates of epitopes on C and P proteins were significantly increased in CHep patients relative to CInf patients. Strikingly, a statistically significant elevation in the number of positive epitopes was observed when ALT nonflare patients shifted into the flare phase. Moreover, S76 identified at baseline was confirmed to be associated with a complete response after 48 weeks of telbivudine therapy. Taken together, we identified several functional cure-related B-cell linear epitopes of chronic HBV infection, and these epitopes may serve as vaccine candidates to elicit neutralizing antibodies to treat HBV infection.

## Introduction

Although an effective preventative vaccine has successfully protected newborns from hepatitis B virus (HBV) infection, approximately 300 million people with persistent positivity of hepatitis B surface antigen (sAg) are at high risk of developing HBV-related liver diseases worldwide (1). Currently, widespread application of interferon and nucleos(t)ide analogs (NUCs) attains significant improvement in restraining HBV replication, decreasing the rates of hepatocellular carcinoma and hepatic failure in patients with chronic hepatitis B (CHB); however, achieving sAg loss as an optimal endpoint is still burdensome and challenging (2, 3). Therapeutic vaccine development is needed to accelerate the goal of “the elimination of viral hepatitis as a public health threat by 2030” proposed by the World Health Organization.

HBV is a member of the family *Hepadnaviridae* with an appropriate 3.2 kb genome encoding four open reading frames, which are translated into the viral surface (S), core (C), polymerase (P), and X proteins; each can elicit specific immune responses against HBV (4). Cumulative data have highlighted the critical role of antibodies specific against the S, C, and P proteins triggered by HBV-specific B cells. The detection of hepatitis B surface antibody (sAb) is associated with virus control and disease resolution, and hepatitis B core antibody (cAb), a marker of past or current HBV exposure, is associated with acute liver damage. Meanwhile, antibodies against the P protein were reported to exert antiviral effects (5–7). The large S protein is further divided into 3 domains, PreS_1_, PreS_2_, and S, in which the chief immunogenic target, “a determinant region” is located (8). Epitope-based therapeutic vaccines and corresponding neutralizing antibodies have shown unique advantages in suppressing virus replication and decreasing sAg levels (9–12). However, the persistent existence of sAg and apparent discrepancies between animal models and clinical patients limit their clinical application. In addition, a recent phase 2, randomized, controlled open-label study showed that a designed therapeutic vaccine targeting HBV-encoded S, C, and X proteins failed to decrease the levels of sAg, despite inducing robust immunologic enhancement (13). Therefore, promising alternative novel epitopes urgently need to be identified as immunotherapeutic targets to eradicate HBV.

Chronic HBV infection is a long-term dynamic process of the interaction between the virus and host immunity. This infection is systematically divided into five phases based on alanine aminotransferase (ALT), HBV DNA level, the presence or absence of hepatitis B e antigen (eAg) and liver inflammation (2). High viremia and antigen load hamper both adaptive and innate immunity (14, 15). Persistent exposure to high sAg loading is associated with a dysfunctional HBV-specific immune response (16, 17). Conversely, patients who achieve sAg loss following antiviral treatment always have a satisfactory off-treatment response and a low incidence of hepatocellular carcinoma (18–20). Hence, it is necessary to explore the dominant B-cell epitopes on HBV proteins among patients in different phases and evaluate the association between dominant epitopes and powerful immune responses or favorable treatment responses. The dominant B-cell epitopes in sAg loss patients may be a more promising candidate to boost the HBV-specific immune response and achieve a functional cure. Unfortunately, to date, there is a lack of comprehensive investigation of B-cell epitopes in patients with different natural histories.

In this study, using a peptide array composed of 15-mer overlapping peptides of HBV-encoded S, C, and P proteins and screening with sAg loss sera, we identified 6 S-specific dominant epitopes (S33, S34, S45, S76, S78, and S89) and one C-specific dominant epitope (C37) that reacted with the majority of sera from patients with a functional cure. These data provide information for developing novel epitope candidates to elicit neutralizing antibodies to treat HBV infection.

## Materials and Methods

### Study Subjects

The participants who provided written informed consent in this study consisted of 4 groups. In the first group, a total of 109 chronic HBV infection patients and 18 sAg-negative healthy individuals with normal ALT levels were enrolled for a cross-sectional study. Sixty-three treatment-naïve patients were classified into eAg-positive chronic infection (eAg^+^CInf, n=21), eAg^+^ chronic hepatitis (eAg^+^CHep, n=21), and eAg^-^CInf (n=21) based on an issued clinical practice guideline (2). Forty-six CHB patients who achieved sAg loss following antiviral treatment were recruited. The second group enrolled six patients who transitioned from the ALT nonflare phase to the flare phase from real-life clinical practice. The third group recruited twelve eAg^+^ CHB patients treated with NUCs and fulfilled the criteria by the stopping rule of APASL from a prospective observational cohort. In this off-treatment cohort, six patients were sustained responders. The other six patients were defined as relapsers who experienced clinical relapse (HBV DNA > 2000 IU/mL and ALT > 2 times the upper limit of normal during follow-up discontinuing therapy). In the fourth group, eAg^+^ CHB patients who participated in a clinical trial of telbivudine (trial number: CLDT600ACN07T) were longitudinally studied. Three telbivudine-treated subjects were defined as the complete response (CR) group (with normal ALT, HBeAg seroconversion, and HBV DNA level < 1000 copies/mL) at week 48, and the other subjects were classified as the noncomplete response (NCR, n=2) group. Patients who suffered from autoimmune diseases, other active diseases, or coinfection with HAV, HCV, HDV, or HIV were excluded. All subjects were recruited at Nanfang Hospital (Guangzhou, China). This study was conducted in compliance with the Declaration of Helsinki and approved by the Ethical Committee of Nanfang Hospital.

### Serological Assays and HBV DNA Assays

The levels of human serum sAg, sAb, eAg, and eAb were quantitatively determined using the Roche COBAS^®^ 6000 analyzer (Roche Molecular Diagnostics, Rotkreuz, Switzerland). The sAg had a lower limit of detection of 0.05 IU/mL. For the cross-sectional cohort, the levels of serum HBV DNA were quantified by the Roche LightCycler^®^ 480 II (Roche Molecular Diagnostics, Pleasanton, CA) with a Hepatitis B Viral Quantitative Fluorescence Diagnostic Kit (Sansure Biotech, Hunan, China), with a lower limit of quantitation of 100 IU/mL. For detection in the longitudinal cohort, serum HBV DNA was quantified by a Roche Cobas Amplicor PCR assay (Roche Molecular Systems, Branchburg, NJ, USA). The detection limit of HBV DNA was no lower than 300 copies/mL. The normal ranges for ALT and AST levels were 9–50 U/L and 15–40 U/L, respectively.

### Phenotype Analysis and Intracellular Cytokine Staining (ICS)

Peripheral blood mononuclear cells (PBMCs) were isolated from fresh heparinized blood by Ficoll-Hypaque density gradient centrifugation, and some cells were cryopreserved in liquid nitrogen for further analysis. After staining with a Live/Dead Fixable Near-IR Dead Cell Stain kit (Life Technologies), thawed PBMCs (5 × 10^5^ cells/tube) were stained with fluorescence phenotype antibodies (CD19-BV510, 562947, BD; CD10-PE-CF594, 562396; CD21-BV421, 562966, BD; CD27-Per-Cy5.5, 560612, BD; CD38-PE-Cy7, 560677, BD; or CD150-BV421, 562875, BD) at 4°C for 30 minutes and analyzed on a BD Aria III flow cytometer (BD Bioscience). To assess the function of B cells, PBMCs were stimulated with PMA (50 ng/mL), ionomycin (0.75 µg/mL), CPG (10 µg/mL, InvivoGen), CD40L (1 µg/mL, PeproTech), and BFA (1 µg/mL). The ICS was performed as previously described (21). Briefly, cells were stained with CD19-BV510, fixed and permeabilized using a Cytofix/Cytoperm kit (BD Bioscience), and then staining was performed with the corresponding intracellular antibody (IFN-γ-PE/Dazzle™ 594, 562875, Biolegend). All flow cytometric analyses were performed using FlowJo V10.0.7 software (Treestar).

### Peptide Array and Serum Screening

Ninety-eight 15-mer peptides overlapping by 11 residues covering large surface (S) proteins (PreS_1_, PreS_2_, and S region) were selected for the assay (**Supplementary Table 1**). In addition, the top response rates of 15-mer peptides in the C protein (top 18) and P protein (top 16) from our previous cultured T-cell enzyme-linked immunospot assay (ELISpot) were also included for the assay (**Supplementary Table 1**). The peptides covering the entire sequence of HBV genotypes B and C were gifts from Johnson & Johnson. The peptide array was manufactured by Suzhou Epitope Biotechnology Co., Ltd. (Suzhou, China). Approximately 0.6 nL of each peptide with a concentration of 0.1 mg/mL was printed onto activated nanomembranes by SmartArrayer 48 as previously described (22, 23). Serum was immediately withdrawn and frozen at -20°C until use. Sera were diluted (1:100) and incubated with a peptide array at 37°C for 30 minutes. Diluted sera without precoated peptide (buffer dot) in each microarray were used as a negative control. After washing, the peptide arrays were incubated with horseradish peroxidase (HRP)-conjugated goat anti-human IgG. Dots were visualized by Super Signal Femto Maximum Sensitivity Substrate for Chemiluminescence (Thermo Fisher), and images were captured by a cool CCD. The mean +3 SD of the buffer dot was used as the background intensity. A signal above 1500 was considered positive. Positive peptide coverage was defined as the ratio of the number of peptides recognized by at least one serum sample to the total number of peptides in each subpartition. The average recognition rate was generated to define the average peptide reaction capacity of patients in subpartitions.

### Statistical Analysis

Data are expressed as median (interquartile range). The Mann–Whitney *U* test or Wilcoxon signed-rank test was used when two groups were compared. For multiple group comparisons, the Kruskal–Wallis H test and *post hoc* test (Dunn’s test) were performed. Correlations between variables were assessed with Spearman’s rank-order correlation coefficient. Categorical variables were compared by the chi-square test. SPSS Statistics 20.0 (Chicago, IL) and GraphPad Prism 8 software were used for statistical analysis. All statistical analyses were based on two-tailed hypothesis tests, and a *P* value < 0.05 was considered statistically significant.

## Results

### B-Cell Subsets Varied in sAg Loss and Were Associated With Successful Treatment Withdrawal

**Figure 1:**
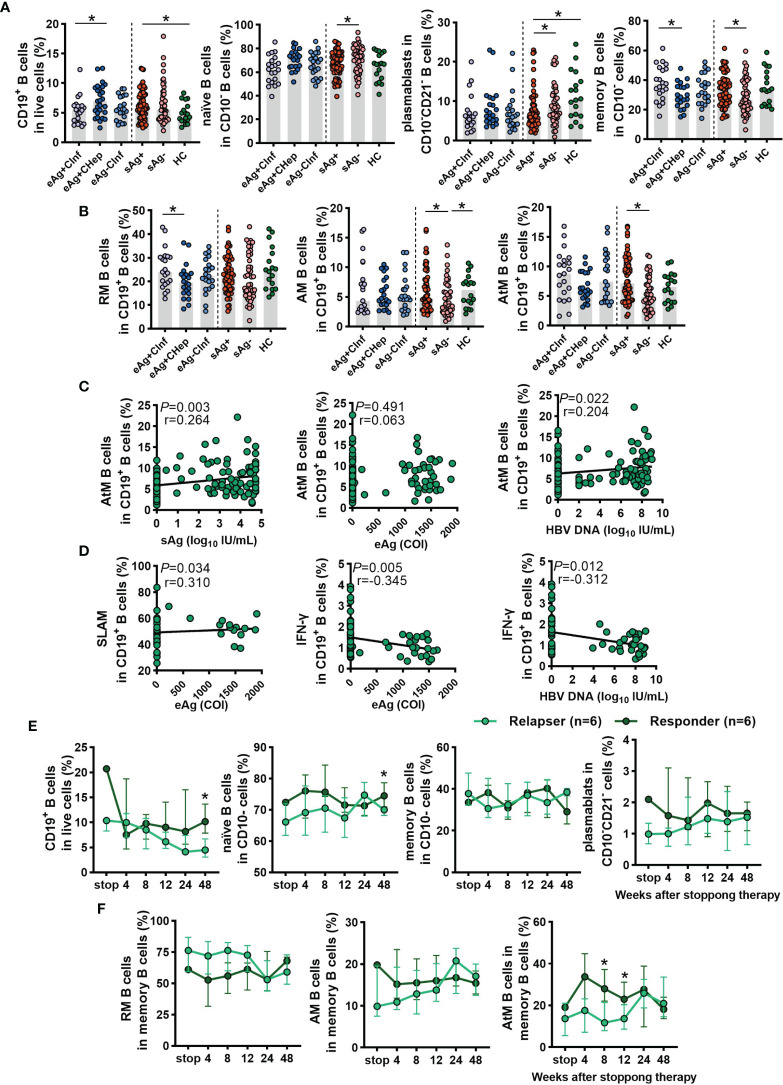
Analyses of the B-cell subsets in the cross-sectional cohort and NUCs withdrawal cohort. **(A)** Comparison of the frequencies of total CD19^+^ B cells, naïve B cells, plasmablasts, and memory B cells in patients with chronic HBV infection and HCs. According to the EASL clinical practice guideline, these subjects were classified into 4 groups: hepatitis B e antigen-positive chronic infection (eAg^+^CInf, n=21), eAg-positive chronic hepatitis (eAg^+^CHep, n=21), eAg^-^CInf (n=21), and hepatitis B surface antigen-negative (sAg-, n=46). **(B)** Frequency of resting memory (RM), activated memory (AM), and atypical memory (AtM) B cells among CD19^+^ B cells in HBV patients and HCs. **(C)** Spearman’s correlation between the frequency of AtM B cells among CD19^+^ B cells and the levels of serum virological parameters. **(D)** The correlation between the expression of SLAM and IFN-γ on CD19^+^ B cells and the levels of serum virological parameters. **(E, F)** Longitudinal analysis of B-cell subsets after stopping NUCs therapy. **(A, B)** Kruskal–Wallis H test and Dunn’s multiple comparisons test. **(C, D)** Spearman’s rank correlation test. **(E, F)** Mann–Whitney *U* test. ^*^ *P* < 0.05.

First, we investigated the percentage of total B cells, naïve B cells, plasmablasts, total memory, resting memory (RM), activated memory (AM), and atypical memory (AtM) B-cell subsets in patients with different immune statuses of HBV infection as well as healthy controls (HCs) (**Table 1** and **Supplementary Figure 1A**). The frequency of total CD19^+^ B cells in PBMCs was significantly higher in sAg^+^ patients than in HCs (**Figure 1A**). The frequency of naïve B cells and plasmablasts was preferentially increased, whereas total memory B cells were significantly reduced in sAg^-^ patients compared with sAg^+^ patients (**Figure 1A**). In addition, a decreased proportion of AM or AtM B cells was observed in patients who achieved sAg loss, and RM B cells were the dominant phenotype in all phases (**Figure 1B** and **Supplementary Figure 1B**). We next examined whether the frequency of AtM B cells was correlated with virological parameters. The frequency of AtM B cells was positively correlated with serum sAg and HBV DNA levels (**Figure 1C**). In addition, a similar positive correlation was detected between the expression of SLAM on CD19^+^ B cells and serum eAg levels. In contrast, inverse correlations were found between IFN-γ-expressing CD19^+^ B cells and eAg and HBV DNA levels (**Figure 1D**). The expression of SLAM on CD19^+^ B cells was similar among all phases, but IFN-γ-expressing CD19^+^ B cells were expanded in eAg^-^CInf patients (**Supplementary Figure 1C**). An increased frequency of CD19^+^ B cells and naïve B cells within the cohort studied was found in responders at week 48 after NUCs discontinuation (**Table 2** and **Figure 1E**). Notably, AtM B cells were dramatically enriched in responders 8 and 12 weeks after stopping treatment compared to relapsers (**Figure 1F**). Together, these results indicated that alteration of the distribution and function of B-cell subsets might be associated with the outcome of HBV infection.

**Table 1:** Clinical characteristics of the cross-sectional study subjects.

Group	eAg+ chronic infection	eAg+ chronic hepatitis	eAg- chronic infection	sAg loss	Healthy controls
No. of patients (M/F)	21 (12/9)	21 (17/4)	21 (14/7)	46 (41/5)	18 (8/10)
Age (year)	30.00 (24.75-36.00)	30.00 (25.50-33.50)	37.00 (33.75-46.00)	41.00 (35.00-53.00)	24.00 (23.00-27.00)
HBV DNA (lg IU/mL)	8.23 (7.78-8.53)	7.91 (7.62-8.24)	2.00^a^	T.N.D	n.d.
ALT (ULN)	0.45 (0.37-0.59)	3.14 (1.90-6.34)	0.40 (0.24-0.53)	0.44 (0.31-0.61)	0.28 (0.18-0.30)
AST (ULN)	0.56 (0.51-0.57)	2.20 (1.23-3.40)	0.50 (0.41-0.64)	0.50 (0.45-0.58)	0.35 (0.37-0.67)
sAg (P/N)	21/0	21/0	21/0	0/46	0/18
sAb (P/N)	0/21	0/21	0/21	22/24	18/0
eAg (P/N)	21/0	21/0	0/21	2/44	0/18
eAb (P/N)	0/21	0/21	21/0	27/19	0/18

Data were shown as median (25-75% percentile).

a Eighteen subjects were lower than 2.0 in HBeAg- chronic infection.

ALT, alanine aminotransferase; AST, aspartate aminotransferase; eAb, hepatitis B e antibody; eAg, hepatitis B e antigen; sAb, hepatitis B surface antibody; sAg, hepatitis B surface antigen; n.d., not determined; P/N, positive or negative; T.N.D, target not detected; ULN, the upper limit of normal.

**Table 2:** Clinical characteristics of responders and relapsers with chronic HBV infection at the end of treatment.

Group	responder	relapser
No. of patients (M/F)	6 (6/0)	6 (5/1)
Age (year)	32.00 (29.25-37.50)	42.00 (37.00-48.25)
ALT (ULN)	0.40 (0.37-0.57)	0.44 (0.36-0.84)
Quantitative sAg (IU/mL)	232.50 (20.91-1787.00)	1553.00 (76.20-5325.00)
eAg (P/N)	0/6	0/6
eAb (P/N)	6/0	6/0
HBV DNA (lg IU/mL)	T.N.D	T.N.D
Median duration of medication (weeks)	188.00 (107.00-264.00)	290.00 (200.00-374.00)
Median time to virological relapse (weeks)	NA	107.00 (12.00-150.00)

Data were shown as median (25-75% percentile).

ALT, alanine aminotransferase; eAb, hepatitis B e antibody; eAg, hepatitis B e antigen; sAg, hepatitis B surface antigen; NA, not applicable; P/N, positive or negative; T.N.D, target not detected; ULN, the upper limit of normal.

### Identification of Dominant S-Specific Linear B-Cell Epitopes of Chronic HBV Infection by a Peptide Array

**Figure 2:**
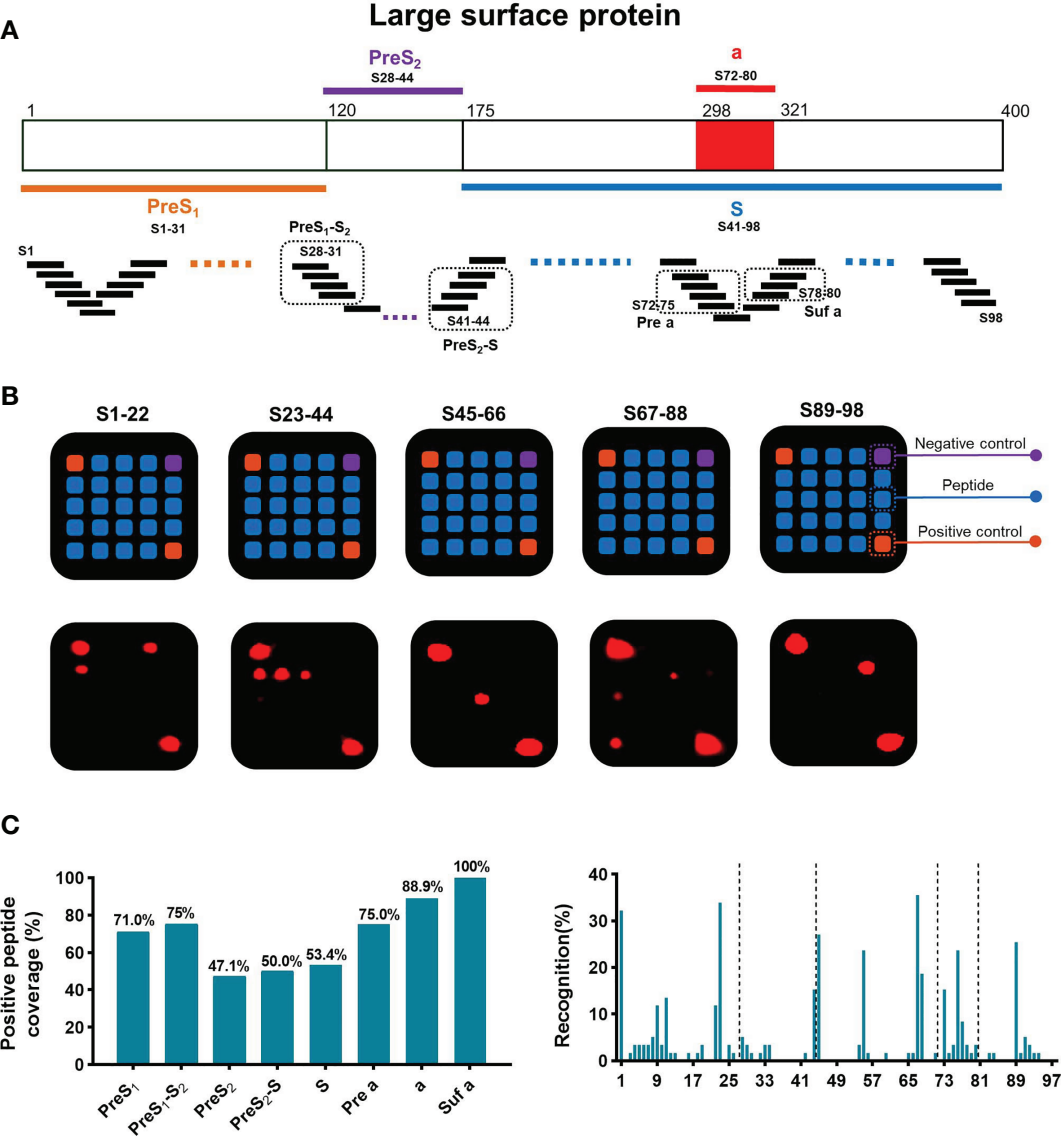
General recognition of linear B-cell epitopes on S protein by peptide array. **(A)** Schematic of the large surface proteins PreS_1_, PreS_2_, and S. In the diagram of S, the “a determinant region” is red. Design of peptide array. Fifteen-mer overlapping peptides covering the entire S protein were shown. According to the classical regions of the large S protein, these peptides were classified into 8 subpartitions: PreS_1_ (S1–31), PreS_1_-S_2_ (S28–31), PreS_2_ (S28–44), PreS_2_-S (S41–44), S (S41–98), Pre a (S72–75), a (S72–80), and Suf a (S78–80). **(B)** Representative peptide array. Design of the 5*5 peptide array; for each subarray, there were negative and positive controls (top). Five representative subarrays with positive results (bottom). **(C)** Positive peptide coverage in subpartitions and recognition rate of each peptide in patients with chronic HBV infection.

Detection of sAbs produced by B cells is associated with disease resolution and virus control in chronic HBV infection. To map the dominant linear B-cell epitopes of sAbs recognized by these patients, a peptide array composed of 98 overlapping 15-mer peptides covering the large S protein was conducted (**Figure 2A**). According to the classical regions of the large S protein, these peptides were classified into 8 subpartitions: PreS_1_ (S1–31), PreS_1_-S_2_ (S28–31), PreS_2_ (S28–44), PreS_2_-S (S41–44), S (S41–98), Pre a (S72–75), a (S72–80), and Suf a (S78–80). Then, we used this peptide array to perform serum screening for a total of 59 serum samples from patients with chronic HBV infection, including 17 sAg loss patients. The design of the 5*5 peptide array and representative results were shown in **Figure 2B**. In summary, the positive peptide coverage of each subpartition varied from 47.1% to 100%, although the recognition rate of each peptide was low (**Figure 2C**).

### sAg Loss Patients Have Fewer B-Cell Linear Epitopes but a Higher Recognition Rate

**Figure 3:**
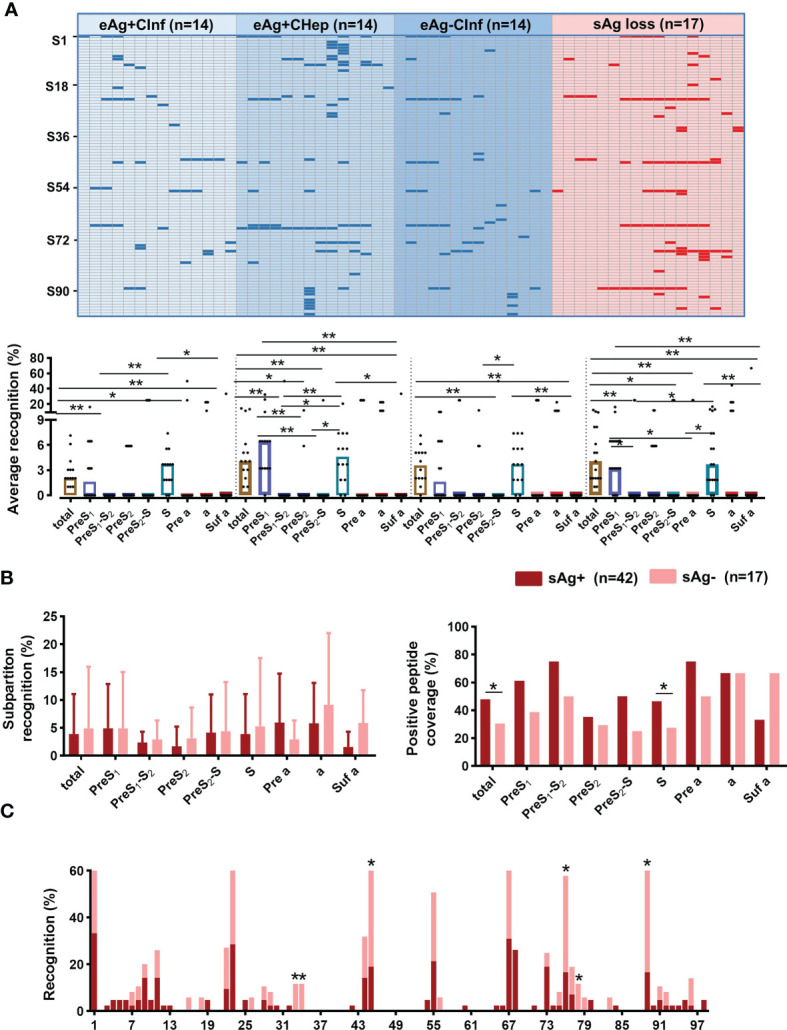
Screening sera from patients with chronic HBV infection. **(A)** Sera from patients with chronic HBV infection were diluted (1:100) and incubated with a peptide array, followed by incubation with HRP-conjugated anti-human IgG. The peptide array was visualized by chemiluminescent reagents, scanned, and quantified. Peptides with a signal above 1500 (mean +3 SD of the negative control) were counted as positive (blue and red bars). The positive peptides for each subject were plotted. Comparing the average recognition rate of each peptide in subpartitions in patients with chronic HBV infection. **(B)** Comparison of the subpartition recognition rate (a set of recognition rates in the subpartition of each patient) and positive peptide coverage between the sAg^+^ and sAg^-^ groups. **(C)** Comparison of the recognition rate of each peptide between sAg^+^ and sAg^-^ groups. **(A)** Kruskal–Wallis H test and Dunn’s multiple comparisons test. **(B)** Mann–Whitney *U* test (left) and Chi-square test (right). **(C)** Chi-square test. ^*^ *P* < 0.05, ^**^ *P* < 0.01.

Then, we attempted to identify the dominant B-cell epitopes shared by patients in different immune phases. The peptide array showed that the dominant linear B-cell epitopes mainly lay in the S subpartition in all stages. An increasing recognition rate of the PreS_1_ subpartition was also observed in eAg^+^CHep and sAg loss patients (**Figure 3A** and **Supplementary Figure 2**). We next compared the recognition rate and positive peptide coverage of subpartitions between patients with sAg^+^ and sAg^-^. The subpartition recognition rate was comparable in these two groups; however, the positive peptide coverage of the total large S protein and S subpartition was lower in sAg^-^ patients (**Figure 3B**). In contrast, six peptides (S33, S34, S45, S76, S78, and S89) reacted with the majority of sera from patients who achieved sAg loss (**Figure 3C**). Overall, these data implied that the dominant linear B-cell epitopes appear to be associated with the prognosis of chronic HBV infection.

### A Specific Linear Epitope on the “A Determinant Region” Identified by sAg Loss Sera Is Associated With a Favorable Treatment Response

**Figure 4:**
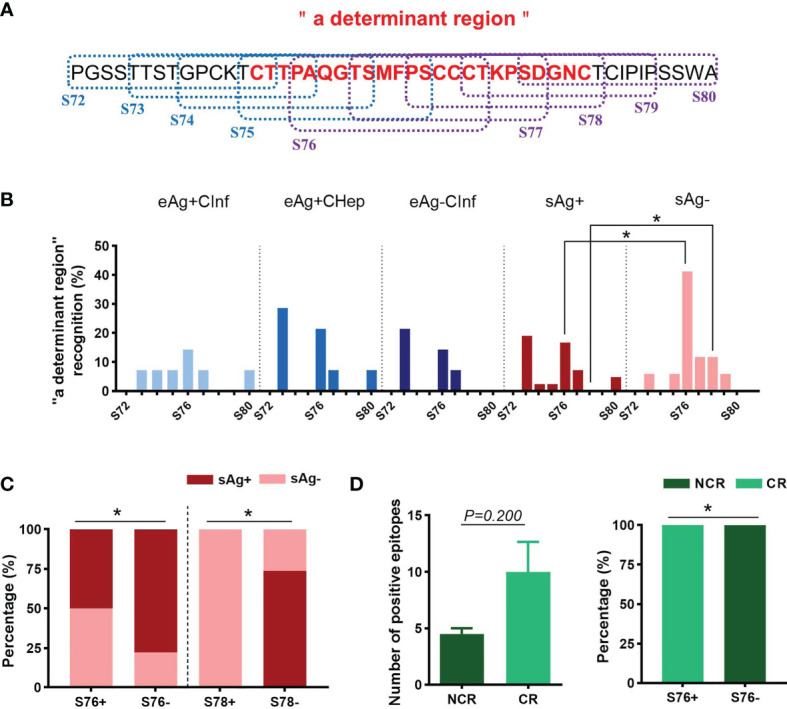
Distribution of linear B-cell epitopes on “a determinant region”. **(A)** The amino acid sequence of “a determinant region”. The dotted boxes indicate the corresponding peptides. The “a determinant region” was in red. **(B)** The recognition rate of each peptide in “a determinant region”. **(C)** The proportion of patients with detectable sAg or sAg loss in the S76+/- or S78+/- groups. **(D)** Comparison of the number of positive peptides between the complete response (CR) and noncomplete response (NCR) groups (left). The proportion of patients with different treatment responses in the S76+/- group (right). **(B–D)** Chi-square test. ^*^ *P* < 0.05.

Considering the pivotal role of antibodies against the “a determinant region” during vaccination, we then investigated the dominant linear B-cell epitopes in “a” subpartition (**Figure 4A**). Relative to patients with sAg^+^, the recognition rates of S76 and S78 were significantly increased in patients with sAg^-^ (**Figure 4B**). In addition, the proportion of sAg^-^ patients in the S76- or S78-positive group was significantly higher than that in the S76- or S78-negative group (**Figure 4C**). Intriguingly, the longitudinal analysis showed an expansive tendency of positive epitopes in patients who achieved complete response (CR) after 48 weeks of telbivudine treatment. In addition, all patients with S76 positivity at baseline achieved CR after therapy (**Table 3** and **Figure 4D**). These results suggested that the S76 linear B-cell epitope may be a good marker to predict a favorable treatment response to telbivudine.

**Table 3:** Clinical characteristics of the study subjects with telbivudine therapy for the longitudinal study.

Group		CR	NCR	*P* value
No. of patients (M/F)		3 (1/2)	2 (2/0)	0.136^a^
Age (year)		34.00 (24.00-35.00)	25.50 (25.00-26.00)	0.800^b^
0 weeks	HBV DNA (lg copies/mL)	8.63 (8.38-8.88)	8.18 (8.04-8.32)	0.800^b^
	ALT (U/L)	253.00 (159.00-256.00)	258.00 (222.00-294.00)	0.983^b^
	eAg (P/N)	3/0	2/0	
48 weeks	HBV DNA (lg copies/mL)	n.d.	n.d.	
	ALT (U/L)	14.00 (10.00-18.00)	19.00 (15.00-23.00)	0.400^b^
	eAg (P/N)	0/3	2/0	0.025^a^

Data were shown as median (25-75% percentile).

a Chi-squared test.

b Mann-Whitney U test.

ALT, alanine aminotransferase; CR, complete response; eAg, hepatitis B e antigen; NCR, non-complete response; P/N, positive or negative.

### Dominant Linear B-Cell Epitopes Are Expanded in Chronic HBV-Infected Patients With Liver Inflammation

**Figure 5:**
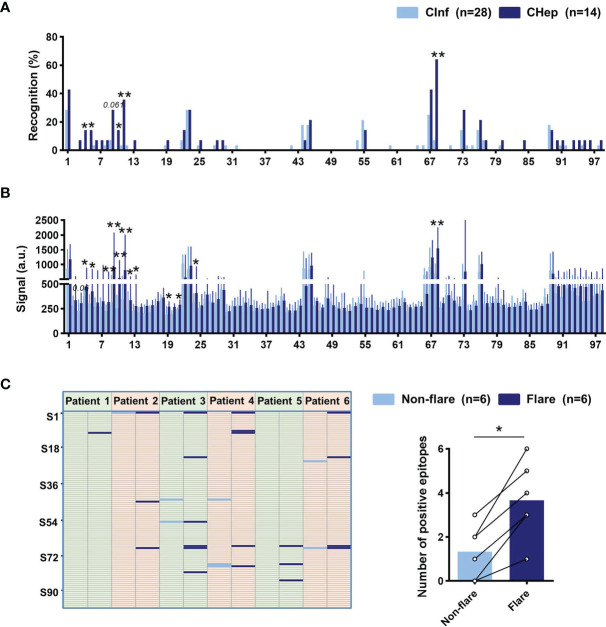
Linear B-cell epitope variation between chronic HBV infection (CInf) and chronic hepatitis B (CHep). **(A)** Comparison of the recognition rate of each peptide between CInf and CHep patients. **(B)** The original signal change of each peptide between CInf and CHep patients. **(C)** Distribution of linear B-cell epitopes of six patients who transitioned from the nonflare eAg^+^CInf phase to the eAg^+^CHep phase with ALT flares. Positive epitopes were plotted in the nonflare (light blue bar) and flare (dark blue bar) phases (left). Comparing the number of positive peptides between ALT nonflare and flare phases (right). **(A)** Chi-square test. **(B)** Mann–Whitney *U* test. **(C)** Wilcoxon signed-rank test. ^*^ *P* < 0.05, ^**^ *P* < 0.01.

Chronic HBV infection is an extremely complicated dynamic process, especially in the hepatitis phase, in which elevated ALT reflects inflammatory activity accompanied by immune activation. We further characterized the distribution of linear B-cell epitopes between patients with CInf and CHep. We found that more linear B-cell epitopes of sAbs were detected in CHep patients, and five linear B-cell epitopes (S4, S5, S10, S11, and S68) were overwhelmingly recognized by CHep patients (**Figure 5A**). Strikingly, the results also showed an elevated intensity of linear epitope signals in CHep patients (**Figure 5B**). Subsequently, we explored linear B-cell epitopes from six eAg^+^CInf patients with normal ALT levels who shifted into the eAg^+^CHep phase with ALT flares during the follow-up. As shown in **Figure 5C**, new epitopes appeared in the flare phase of these patients. Of note, there was a statistically significant elevation in the number of positive epitopes when patients shifted into the ALT flare phase (**Figure 5C**). Collectively, these results showed that dominant linear B-cell epitopes could reflect immune activation in CHB.

### A Higher Linear B-Cell Epitope Recognition Rate on C and P Proteins in CHep

**Figure 6:**
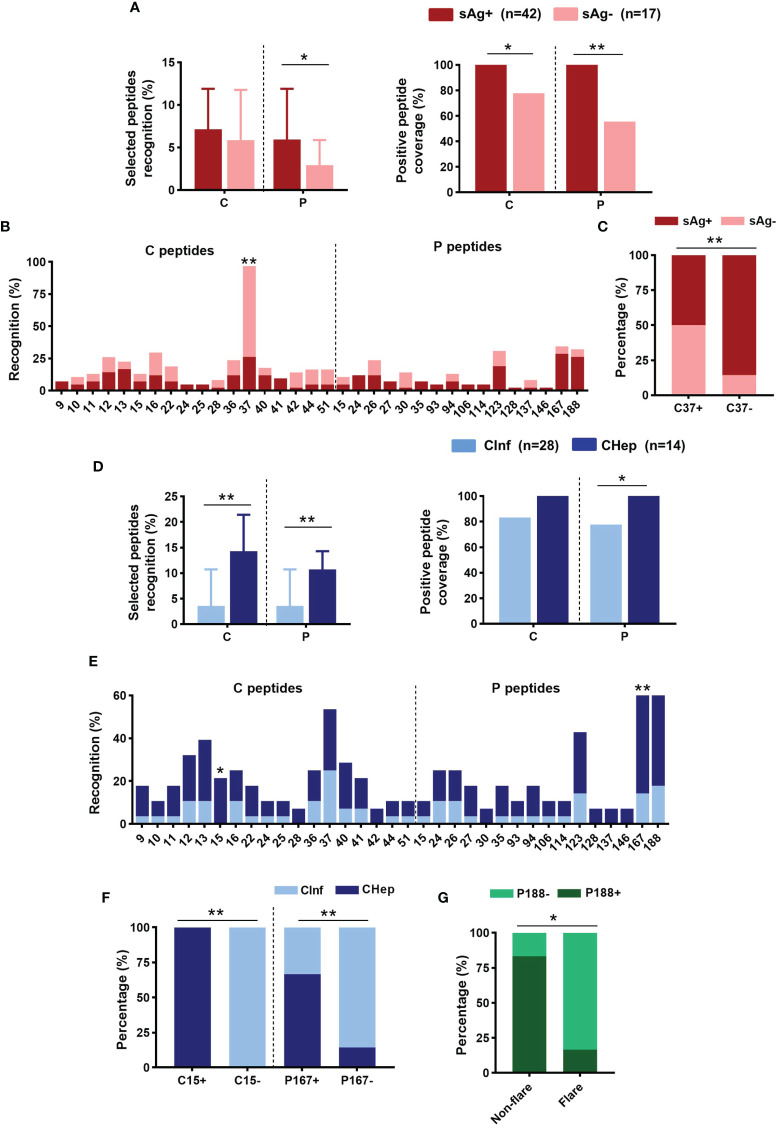
Distribution of linear B-cell epitopes on core (C) and polymerase (P) proteins. **(A)** Comparison of the recognition rate and positive peptide coverage on C or P between sAg^+^ and sAg^-^ groups. **(B)** The recognition rate of the selected peptide on C or P. **(C)** Proportion of patients with detectable sAg or sAg loss in the C37+/- group. **(D)** Comparison of the recognition rate and positive peptide coverage on C or P proteins between CInf and CHep patients. **(E)** Comparison of the recognition rate of the selected peptides between CInf and CHep patients. **(F)** The proportion of patients with CInf or CHep in the C15+/- or P167+/- groups. **(G)** The proportion of P188 was positive or negative in the ALT nonflare and flare phases of those six patients. **(A, D)** Mann–Whitney *U* test (left) and Chi-square test (right). **(B, C, E–G)** Chi-square test. ^*^ *P* < 0.05, ^**^ *P* < 0.01.

Additionally, we tested the dominant linear B-cell epitopes of the C and P proteins in chronic HBV infection. The top 18 response rates of 15-mer peptides in the C protein and the top 16 in the P protein from our earlier cultured T-cell ELISpot were selected, and all can be identified in the array (**Supplementary Figures 3A, B**). An increasing number of positive epitopes on the P protein were shown in eAg^+^CHep patients relative to patients with sAg loss (**Supplementary Figure 3C**). Patients carrying sAg displayed a higher recognition rate of the selected peptides on the P protein than those with sAg^-^; correspondingly, the positive peptide coverage of the C and P proteins in sAg^+^ patients was higher than that in sAg^-^ patients (**Figure 6A**). Strikingly, C37 on the C protein stood out from the selected peptides; its recognition rate was significantly increased in patients with sAg^-^; moreover, the proportion of sAg^-^ patients in the C37-positive group was significantly higher than that in the C37-negative group (**Figures 6B, C**). We also examined the variation in linear B-cell epitopes between CInf patients and CHep patients. A higher recognition rate of the selected peptides on C and P proteins was observed in CHep patients, along with higher positive peptide coverage (**Figure 6D**). Compared with CInf patients, the recognition rate of C15 on C protein and P167 on P protein were significantly increased in CHep patients (**Figure 6D**). The proportion of CHep patients in the C15- or P167-positive group was significantly higher than that in the C15- or P167-negative group (**Figures 6E, F**). Notably, a remarkable decrease in the proportion of P188 was found in the flare phase compared to the nonflare stage in these six patients during follow-up (**Figure 6G**). These findings indicated a broad value of linear B-cell epitopes on HBV proteins to evaluate the prognoses of patients.

## Discussion

Previous studies have identified several neutralizing antibodies against HBV protein from a small number of HBV vaccinees or controllers (9, 24). In contrast, we screened sera from 59 patients with chronic HBV infection, including 17 patients who achieved sAg loss by a peptide array. The same strategy has been used to identify dominant epitopes in other infectious diseases (22, 23, 25, 26). To learn the profile of the B-cell linear epitopes recognized by chronic HBV infection for guiding vaccine design, we devised a peptide array composed of 15-mer overlapping peptides of HBV-encoded S, C, and P proteins and performed a screening on B-cell linear epitopes with sera from patients in different phases of the natural history. The data presented in this study provide information for developing novel epitope candidates to potentially elicit neutralizing antibodies to treat chronic HBV infection. First, the proportion of dysfunctional AtM B cells was decreased in patients who achieved sAg loss and was associated with successful treatment withdrawal. Second, we identified seven dominant epitopes recognized by sAg loss patients (S33, S34, S45, S76, S78, S89, and C37), and a specific epitope, S76, at baseline was associated with a favorable treatment response to telbivudine therapy. Third, dominant linear B-cell epitopes are expanded in chronic hepatitis B. Future studies are needed to further comprehend the role and neutralization capacity of antibodies against these epitopes.

Clinically, sAg is a sensitive diagnostic marker for HBV infection. Serum sAg titers are highly correlated with reactivity to antiviral therapy and prognosis. Moreover, the incapacitated immune system induced by overwhelming sAg was the real culprit for the unsatisfactory effect of anti-HBV therapy (27, 28). Therefore, sAg is becoming the most promising target for epitope-based therapy to achieve a functional cure. Three forms of HBV S protein were displayed on HBV virions: the large S (PreS_1_+PreS_2_+S), the middle S (PreS_2_+S), and the small S (S). Antibodies against the prime target “a determinant region” in the S domain, the antigenic loop and central immunodominant region for HBV prevention and therapy, conferred efficient viremia suppression: E6F6-like mAbs targeted epitope on “a determinant region” (recognize aa 119–125 of S) strikingly suppressed HBV DNA and HBsAg levels in an HBV mouse model (10). To attain more aggressive HBV-specific immune responses and therapeutic effects, they further designed S-aa 119–125-containing B cell epitope-based therapeutic vaccines. The remarkable and prolonged suppression effects on sAg and viral loading in HBV carrier mice confirmed its potential in therapeutic vaccine design for CHB treatment (11). However, antibodies against a similar recognition site were not found in a human counterpart (12). In the present study, we demonstrated that the recognition rates of S76 and S78 on “a determinant region” were distinctly higher in sAg loss patients despite decreased positive peptide coverage of the S domain. Total memory B cells and AtM B cells, the main component of sAg-specific B cells (29–31), were reduced in sAg loss, which mainly resulted from the decline of sAg and eAg. Combined with the findings that an increasing proportion of plasmablasts in sAg loss could differentiate into antibody-producing cells and an inverse association between the frequency of immunoregulatory IFN-γ-producing B cells and virological parameters, it is reasonable to speculate that fewer defective B cells in sAg loss may produce effective neutralizing antibodies against these two epitopes in conquering sAg seroclearance. To further investigate the relationship between these epitopes and treatment response, we longitudinally analyzed the dominant linear B-cell epitope in patients treated with telbivudine for 48 weeks. Notably, patients identified as S76, S1, or S9 at baseline were more likely to achieve CR after the entire therapy (**Figure 4D** and **Supplementary Figure 4**). It should be emphasized that small quantity of longitudinal participants and patients with sAg loss might cause potential bias, which makes it difficult to match all comparisons; further investigation is warranted to verify this preliminary conclusion.

Recently, an increasing number of publications identified several epitopes besides S76 or S78 that play a pivotal role in inducing the HBV-specific immune response. Further *in vivo* preclinical experiments also confirmed the neutralizing ability of epitope-corresponding antibodies (9, 12, 32). However, the persistent existence of sAg restricted their clinical application. The discrepancies possibly resulted from the various natural histories of selected individuals. In this study, we focused on pursuing candidate epitopes that drove the transformation to sAg seroclearance. The straightforward comparison between sAg loss and sAg-positive patients may make it easier to identify the pivotal epitopes that may play critical roles in achieving a functional cure while neglected under various alternative epitopes in other studies. Of note, the dominant epitopes on the C protein in patients who achieved a functional cure were also consistent with our previous finding that HBV C-specific T-cell responses played an essential role in HBV control (33). To our knowledge, few studies have documented sAg loss-related dominant epitopes, which would have potentially far-reaching ramifications for immunotherapy of chronic HBV infection. Further experiments are needed to clarify this hypothesis, and the therapeutic effect of antibodies against nominated epitopes remains to be elucidated.

Dozens of resources and efforts have been devoted to developing anti-HBV therapy; nevertheless, progress is still tortuous due to the difficulty of overcoming immune tolerance in chronic HBV infection. The natural history of chronic HBV infection is a long-term dynamic process and consists of five discontinuous phases. HBV-specific adaptive immunity can also change from immune tolerance to progressive immune activation, inactivation, reactivation, and exhaustion (34). Emerging evidence has shown that “immune tolerant” CInf patients tend to develop hepatocellular carcinoma compared with “immune active” CHep patients (12% *vs.* 6% per 10 years) (34, 35). In addition, the diverse immune status may be a consequence of the abundance of dominant epitopes, which may strongly influence immune activation signaling (36). Hence, it is essential to explore the epitope variation between CInf and CHep patients. Our peptide array data demonstrated that S4, S5, S10, S11, S68, C15, and P167 were the most distinguishable dominant epitopes in CHep patients relative to CInf patients. Additionally, we longitudinally analyzed the dynamic variation of epitopes in six patients who transitioned from the ALT nonflare eAg^+^CInf phase to the flare eAg^+^CHep phase. Emerging epitopes in the ALT flare phase of these patients further confirmed that dominant linear B-cell epitopes could reflect immune activation in CHB. Interestingly, we found that the majority of dominant epitopes in immune-active CHep patients were located in the PreS_1_ domain. Antibodies against S regions are believed to elicit neutralizing infectivity. Meanwhile, the PreS_1_ domain interacts with the HBV receptor NTCP on hepatocytes (37, 38). Thus, it is reasonable to speculate that the abundance of epitopes reflecting immune activation during chronic HBV infection may activate the HBV-specific immune response, elicit neutralizing antibodies, inhibit virus replication, and disrupt susceptibility to NTCP for HBV entry, which should be elucidated in future studies.

In summary, we identified the dominant B-cell linear epitopes of chronic HBV infection by a peptide array. These results will be essential to guide the therapeutic vaccine design for chronic HBV infection.

## Data Availability Statement

The raw data supporting the conclusions of this article will be made available by the authors, without undue reservation.

## Ethics Statement 

The studies involving human participants were reviewed and approved by Ethical Committee of Nanfang Hospital. The patients/participants provided their written informed consent to participate in this study.

## Author Contributions

SG, LT, and YL designed the study. SG, ZL, and LL performed the experiments and analyses. SG, ZL, SZ, YM, XL, GY, and CW collected samples and laboratory data. SG, ZL, LT, and YL wrote the manuscript. LT and YL supervised the study. All authors contributed to the article and approved the submitted version.

## Funding

This work was supported by grants from the National Natural Science Foundation of China (81971933 and 81770592), National Science and Technology Major Project of China (2018ZX10301202), and the Outstanding Youth Development Scheme of Nanfang Hospital, Southern Medical University (2020J003).

## Conflict of Interest

The authors declare that the research was conducted in the absence of any commercial or financial relationships that could be construed as a potential conflict of interest.

## Publisher’s Note

All claims expressed in this article are solely those of the authors and do not necessarily represent those of their affiliated organizations, or those of the publisher, the editors and the reviewers. Any product that may be evaluated in this article, or claim that may be made by its manufacturer, is not guaranteed or endorsed by the publisher.

## Supplementary Material

Supplementary Table 1 The amino acid sequences of HBV peptides.

Supplementary Figure 1 Cross-sectional analysis of B-cell subsets. **(A)** Gating strategy for total CD19^+^ B cells and their distribution into naïve B cells (CD19^+^CD10^-^CD21^+^CD27^-^), memory B cells (CD19^+^CD10^-^CD21^-^ and CD19^+^CD10^-^CD21^+^CD27^+^), plasmablasts (CD19^+^CD10^-^CD21^-^CD27^+^CD38^+^), resting memory (RM, CD21^+^CD27^+^), activated memory (AM, CD21^-^CD27^+^), and atypical memory (AtM, CD21^-^CD27^-^) B-cell subsets. **(B)** Frequency of RM, AM, and AtM B cells among CD19^+^ B cells in patients with chronic HBV infection. **(C)** The expression of SLAM and IFN-γ on CD19^+^ B cells within patients with chronic HBV infection and HCs. **(B, C)** Kruskal–Wallis H test and Dunn’s multiple comparisons test. **P* < 0.05, ***P* < 0.01.

Supplementary Figure 2 Comparing the number of positive epitopes in patients with chronic HBV infection in different subpartitions.

Supplementary Figure 3 General recognition of linear B-cell epitopes on C and P proteins. **(A)** The recognition rate of the selected peptide on C and P proteins in patients with chronic HBV infection. **(B)** Positive peptide coverage on C and P. **(C)** Comparison of the number of positive epitopes on the C or P protein in patients with chronic HBV infection. **(B)** Chi-square test. **(C)** Mann–Whitney *U* test. **P* < 0.05.

Supplementary Figure 4 The proportion of patients with different treatment responses in the S1+/- or S9+/- groups. Chi-square test. **P* < 0.05.

## References

[1] Polaris Observatory Collaborators. Global Prevalence, Treatment, and Prevention of Hepatitis B Virus Infection in 2016: A Modelling Study. Lancet Gastroenterol Hepatol (2018) 3:383–403. doi: 10.1016/s2468-1253(18)30056-6 29599078

[2] European Association for the Study of the Liver. EASL 2017 Clinical Practice Guidelines on the Management of Hepatitis B Virus Infection. J Hepatol (2017) 67:370–98. doi: 10.1016/j.jhep.2017.03.021 28427875

[3] Fanning GC Zoulim F Hou J Bertoletti A. Therapeutic Strategies for Hepatitis B Virus Infection: Towards a Cure. Nat Rev Drug Discov (2019) 18:827–44. doi: 10.1038/s41573-019-0037-0 31455905

[4] Gerlich WH. Medical Virology of Hepatitis B: How it Began and Where We are Now. Virol J (2013) 10:239. doi: 10.1186/1743-422X-10-239 23870415 PMC3729363

[5] Hoofnagle JH Gerety RJ Barker LF. Antibody to Hepatitis-B-Virus Core in Man. Lancet (1973) 2:869–73. doi: 10.1016/s0140-6736(73)92004-7 4126916

[6] Farci P Diaz G Chen Z Govindarajan S Tice A Agulto L. B Cell Gene Signature With Massive Intrahepatic Production of Antibodies to Hepatitis B Core Antigen in Hepatitis B Virus-Associated Acute Liver Failure. Proc Natl Acad Sci USA (2010) 107:8766–71. doi: 10.1073/pnas.1003854107 PMC2889296 20421498

[7] Hu S Xiong H Kang X Wang S Zhang T Yuan Q. Preparation and Functional Evaluation of Monoclonal Antibodies Targeting Hepatitis B Virus Polymerase. Virulence (2021) 12:188–94. doi: 10.1080/21505594.2020.1869391 PMC7834045 33356842

[8] Urban S Bartenschlager R Kubitz R Zoulim F. Strategies to Inhibit Entry of HBV and HDV Into Hepatocytes. Gastroenterology (2014) 147:48–64. doi: 10.1053/j.gastro.2014.04.030 24768844

[9] Hehle V Beretta M Bourgine M Ait-Goughoulte M Planchais C Morisse S. Potent Human Broadly Neutralizing Antibodies to Hepatitis B Virus From Natural Controllers. J Exp Med (2020) 217:e20200840. doi: 10.1084/jem.20200840 32579155 PMC7537403

[10] Zhang TY Yuan Q Zhao JH Zhang YL Yuan LZ Lan Y. Prolonged Suppression of HBV in Mice by a Novel Antibody That Targets a Unique Epitope on Hepatitis B Surface Antigen. Gut (2016) 65:658–71. doi: 10.1136/gutjnl-2014-308964 26423112

[11] Zhang TY Guo XR Wu YT Kang XZ Zheng QB Qi RY. A Unique B Cell Epitope-Based Particulate Vaccine Shows Effective Suppression of Hepatitis B Surface Antigen in Mice. Gut (2020) 69:343–54. doi: 10.1136/gutjnl-2018-317725 PMC6984059 30926653

[12] Wang Q Michailidis E Yu Y Wang Z Hurley AM Oren DA. A Combination of Human Broadly Neutralizing Antibodies Against Hepatitis B Virus HBsAg With Distinct Epitopes Suppresses Escape Mutations. Cell Host Microbe (2020) 28:335–49.e6. doi: 10.1016/j.chom.2020.05.010 32504577 PMC8182833

[13] Boni C Janssen HLA Rossi M Yoon SK Vecchi A Barili V. Combined GS-4774 and Tenofovir Therapy Can Improve HBV-Specific T-Cell Responses in Patients With Chronic Hepatitis. Gastroenterology (2019) 157:227–41.e7. doi: 10.1053/j.gastro.2019.03.044 30930022

[14] Shin EC Sung PS Park SH. Immune Responses and Immunopathology in Acute and Chronic Viral Hepatitis. Nat Rev Immunol (2016) 16:509–23. doi: 10.1038/nri.2016.69 27374637

[15] Tsai KN Kuo CF Ou JJ. Mechanisms of Hepatitis B Virus Persistence. Trends Microbiol (2018) 26:33–42. doi: 10.1016/j.tim.2017.07.006 28823759 PMC5741523

[16] Fisicaro P Barili V Rossi M Montali I Vecchi A Acerbi G. Pathogenetic Mechanisms of T Cell Dysfunction in Chronic HBV Infection and Related Therapeutic Approaches. Front Immunol (2020) 11:849. doi: 10.3389/fimmu.2020.00849 32477347 PMC7235343

[17] Kim JH Ghosh A Ayithan N Romani S Khanam A Park JJ. Circulating Serum HBsAg Level is a Biomarker for HBV-Specific T and B Cell Responses in Chronic Hepatitis B Patients. Sci Rep (2020) 10:1835. doi: 10.1038/s41598-020-58870-2 32020034 PMC7000714

[18] Kim GA Lim YS An J Lee D Shim JH Kim KM. HBsAg Seroclearance After Nucleoside Analogue Therapy in Patients With Chronic Hepatitis B: Clinical Outcomes and Durability. Gut (2014) 63:1325–32. doi: 10.1136/gutjnl-2013-305517 24162593

[19] Yip TC Wong GL Chan HL Tse YK Lam KL Lui GC. HBsAg Seroclearance Further Reduces Hepatocellular Carcinoma Risk After Complete Viral Suppression With Nucleos(T)Ide Analogues. J Hepatol (2019) 70:361–70. doi: 10.1016/j.jhep.2018.10.014 30367899

[20] Wu S Luo W Wu Y Chen H Peng J. HBsAg Quantification Predicts Off-Treatment Response to Interferon in Chronic Hepatitis B Patients: A Retrospective Study of 250 Cases. BMC Gastroenterol (2020) 20:121. doi: 10.1186/s12876-020-01263-6 32316928 PMC7171920

[21] Ma SW Huang X Li YY Tang LB Sun XF Jiang XT. High Serum IL-21 Levels After 12 Weeks of Antiviral Therapy Predict HBeAg Seroconversion in Chronic Hepatitis B. J Hepatol (2012) 56:775–81. doi: 10.1016/j.jhep.2011.10.020 22173154

[22] Lu Y Li Z Teng H Xu H Qi S He J. Chimeric Peptide Constructs Comprising Linear B-Cell Epitopes: Application to the Serodiagnosis of Infectious Diseases. Sci Rep (2015) 5:13364. doi: 10.1038/srep13364 26293607 PMC4543967

[23] Xue Q Xu H Liu H Pan J Yang J Sun M. Epitope-Containing Short Peptides Capture Distinct IgG Serodynamics That Enable Differentiating Infected From Vaccinated Animals for Live-Attenuated Vaccines. J Virol (2020) 94:e01573–19. doi: 10.1128/JVI.01573-19 PMC7158722 31896600

[24] Wang W Sun L Li T Ma Y Li J Liu Y. A Human Monoclonal Antibody Against Small Envelope Protein of Hepatitis B Virus With Potent Neutralization Effect. mAbs (2015) 8:468–77. doi: 10.1080/19420862.2015.1134409 PMC4966830 26713590

[25] Zhang H Song Z Yu H Zhang X Xu S Li Z. Genome-Wide Linear B-Cell Epitopes of Enterovirus 71 in a Hand, Foot and Mouth Disease (HFMD) Population. J Clin Virol (2018) 105:41–8. doi: 10.1016/j.jcv.2018.06.001 29886372

[26] Yi Z Ling Y Zhang X Chen J Hu K Wang Y. Functional Mapping of B-Cell Linear Epitopes of SARS-CoV-2 in COVID-19 Convalescent Population. Emerg Microbes Infect (2020) 9:1988–96. doi: 10.1080/22221751.2020.1815591 PMC7534331 32844713

[27] Mak LY Seto WK Fung J Yuen MF. Use of HBsAg Quantification in the Natural History and Treatment of Chronic Hepatitis B. Hepatol Int (2020) 14:35–46. doi: 10.1007/s12072-019-09998-5 31745711

[28] Hoogeveen RC Boonstra A. Checkpoint Inhibitors and Therapeutic Vaccines for the Treatment of Chronic HBV Infection. Front Immunol (2020) 11:401. doi: 10.3389/fimmu.2020.00401 32194573 PMC7064714

[29] Burton AR Pallett LJ McCoy LE Suveizdyte K Amin OE Swadling L. Circulating and Intrahepatic Antiviral B Cells are Defective in Hepatitis B. J Clin Invest (2018) 128:4588–603. doi: 10.1172/jci121960 PMC6159997 30091725

[30] Le Bert N Salimzadeh L Gill US Dutertre CA Facchetti F Tan A. Comparative Characterization of B Cells Specific for HBV Nucleocapsid and Envelope Proteins in Patients With Chronic Hepatitis B. J Hepatol (2020) 72:34–44. doi: 10.1016/j.jhep.2019.07.015 31348999

[31] Salimzadeh L Le Bert N Dutertre CA Gill US Newell EW Frey C. PD-1 Blockade Partially Recovers Dysfunctional Virus-Specific B Cells in Chronic Hepatitis B Infection. J Clin Invest (2018) 128:4573–87. doi: 10.1172/jci121957 PMC6159957 30084841

[32] Yato K Onodera T Matsuda M Moriyama S Fujimoto A Watashi K. Identification of Two Critical Neutralizing Epitopes in the Receptor Binding Domain of Hepatitis B Virus Pres1. J Virol (2020) 95:e01680–20. doi: 10.1128/JVI.01680-20. 10.1128/JVI.01680-20. PMC8092832 33298539

[33] Chen C Jiang X Liu X Guo L Wang W Gu S. Identification of the Association Between HBcAg-Specific T Cell and Viral Control in Chronic HBV Infection Using a Cultured ELISPOT Assay. J Leukoc Biol (2021) 109:455–65. doi: 10.1002/JLB.5MA0620-023RR 32620046

[34] Chen Y Tian Z. HBV-Induced Immune Imbalance in the Development of HCC. Front Immunol (2019) 10:2048. doi: 10.3389/fimmu.2019.02048 31507621 PMC6718466

[35] Kim GA Lim YS Han S Choi J Shim JH Kim KM. High Risk of Hepatocellular Carcinoma and Death in Patients With Immune-Tolerant-Phase Chronic Hepatitis B. Gut (2018) 67:945–52. doi: 10.1136/gutjnl-2017-314904 29055908

[36] Kuipery A Gehring AJ Isogawa M. Mechanisms of HBV Immune Evasion. Antiviral Res (2020) 179:104816. doi: 10.1016/j.antiviral.2020.104816 32387476

[37] Yan H Zhong G Xu G He W Jing Z Gao Z. Sodium Taurocholate Cotransporting Polypeptide is a Functional Receptor for Human Hepatitis B and D Virus. eLife (2012) 1:e00049. doi: 10.7554/eLife.00049 23150796 PMC3485615

[38] Bertoletti A Ferrari C. Adaptive Immunity in HBV Infection. J Hepatol (2016) 64:S71–83. doi: 10.1016/j.jhep.2016.01.026 27084039

