# Supplementary figures and images for: Identification and Mapping of HBsAg Loss-Related B-Cell Linear Epitopes in Chronic HBV Patients by Peptide Array
Source: Front Immunol. 2021 Oct 15;12:767000. doi: 10.3389/fimmu.2021.767000 (PMC8554339; doi:10.3389/fimmu.2021.767000)

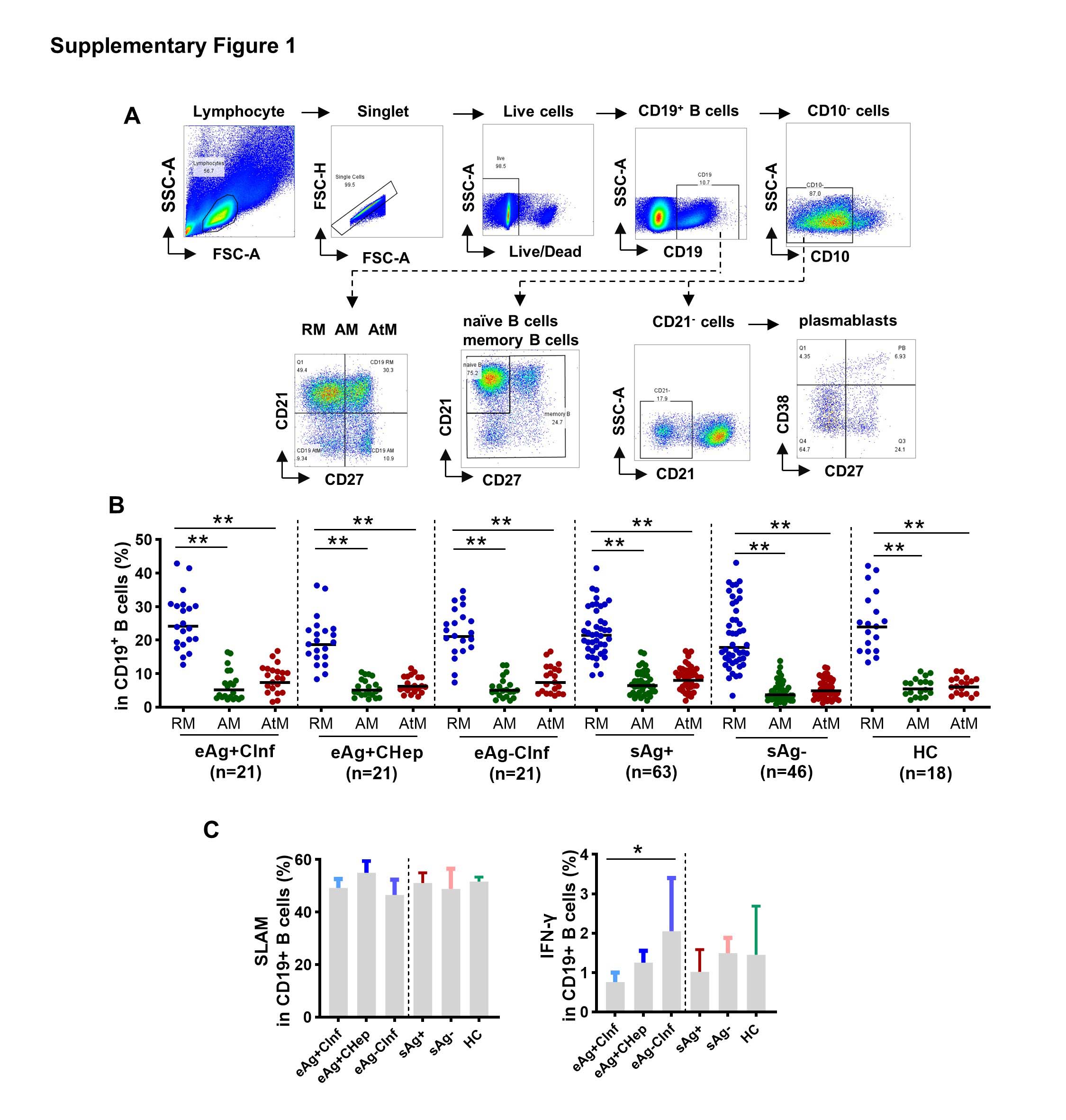

Supplement: Supplementary Figure 1 — Cross-sectional analysis of B-cell subsets. (A) Gating strategy for total CD19+ B cells and their distribution into naïve B cells (CD19+CD10-CD21+CD27-), memory B cells (CD19+CD10-CD21- and CD19+CD10-CD21+CD27+), plasmablasts (CD19+CD10-CD21-CD27+CD38+), resting memory (RM, CD21+CD27+), activated memory (AM, CD21-CD27+), and atypical memory (AtM, CD21-CD27-) B-cell subsets. (B) Frequency of RM, AM, and AtM B cells among CD19+ B cells in patients with chronic HBV infection. (C) The expression of SLAM and IFN-γ on CD19+ B cells within patients with chronic HBV infection and HCs. (B, C) Kruskal–Wallis H test and Dunn’s multiple comparisons test. *P < 0.05, **P < 0.01. [file Image_1.jpeg]

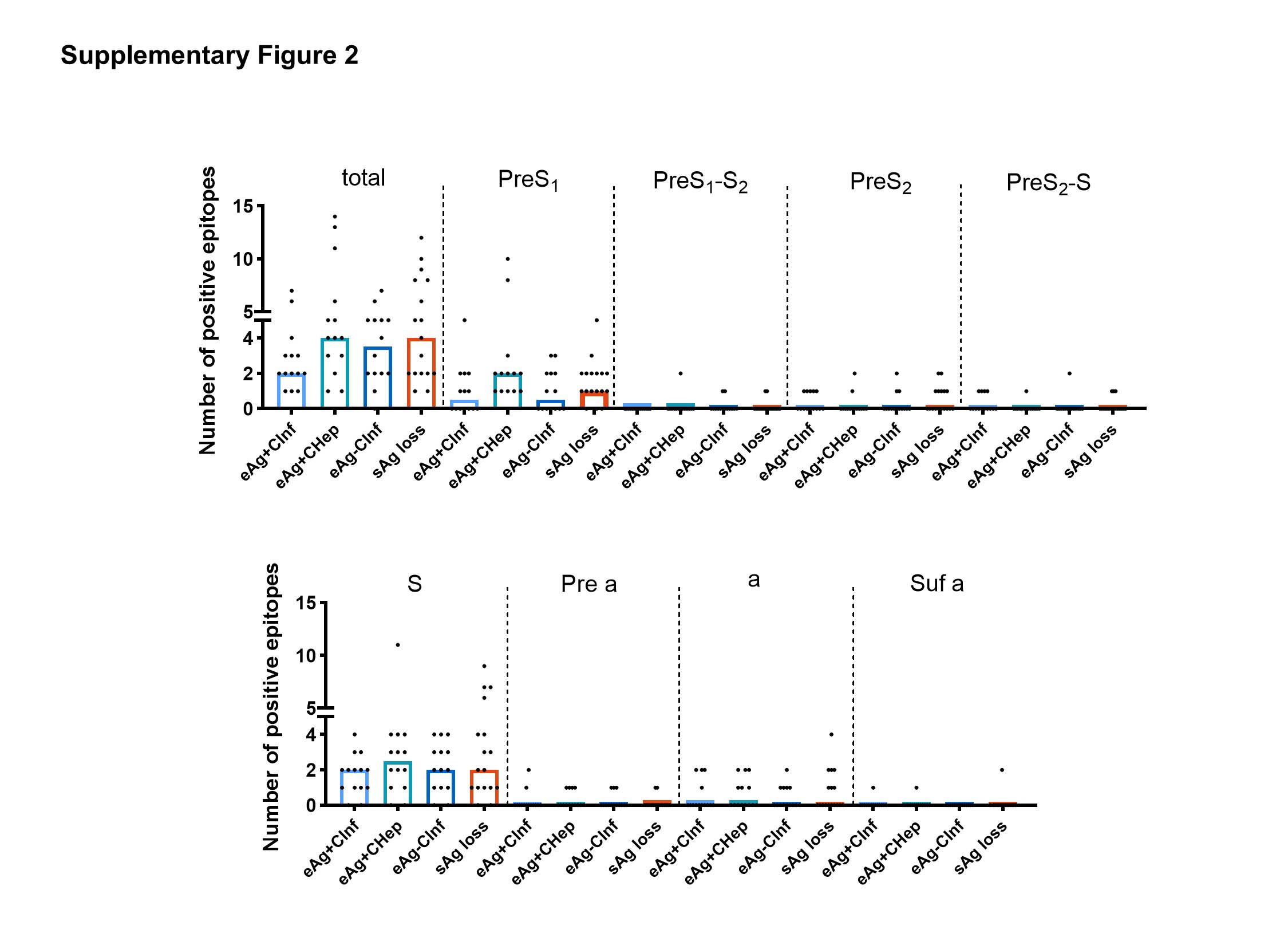

Supplement: Supplementary Figure 2 — Comparing the number of positive epitopes in patients with chronic HBV infection in different subpartitions. [file Image_2.jpeg]

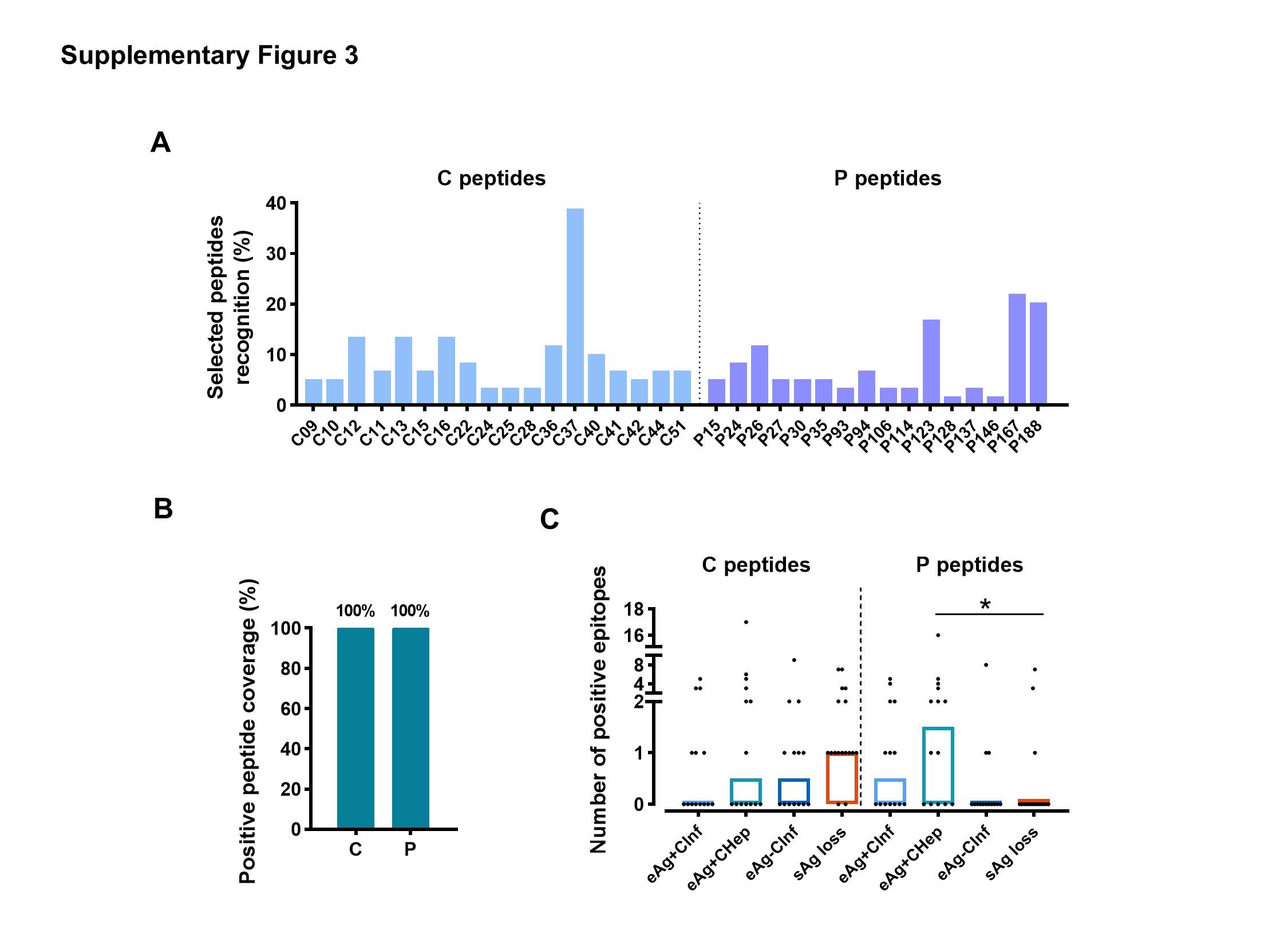

Supplement: Supplementary Figure 3 — General recognition of linear B-cell epitopes on C and P proteins. (A) The recognition rate of the selected peptide on C and P proteins in patients with chronic HBV infection. (B) Positive peptide coverage on C and P. (C) Comparison of the number of positive epitopes on the C or P protein in patients with chronic HBV infection. (B) Chi-square test. (C) Mann–Whitney U test. *P < 0.05. [file Image_3.jpeg]

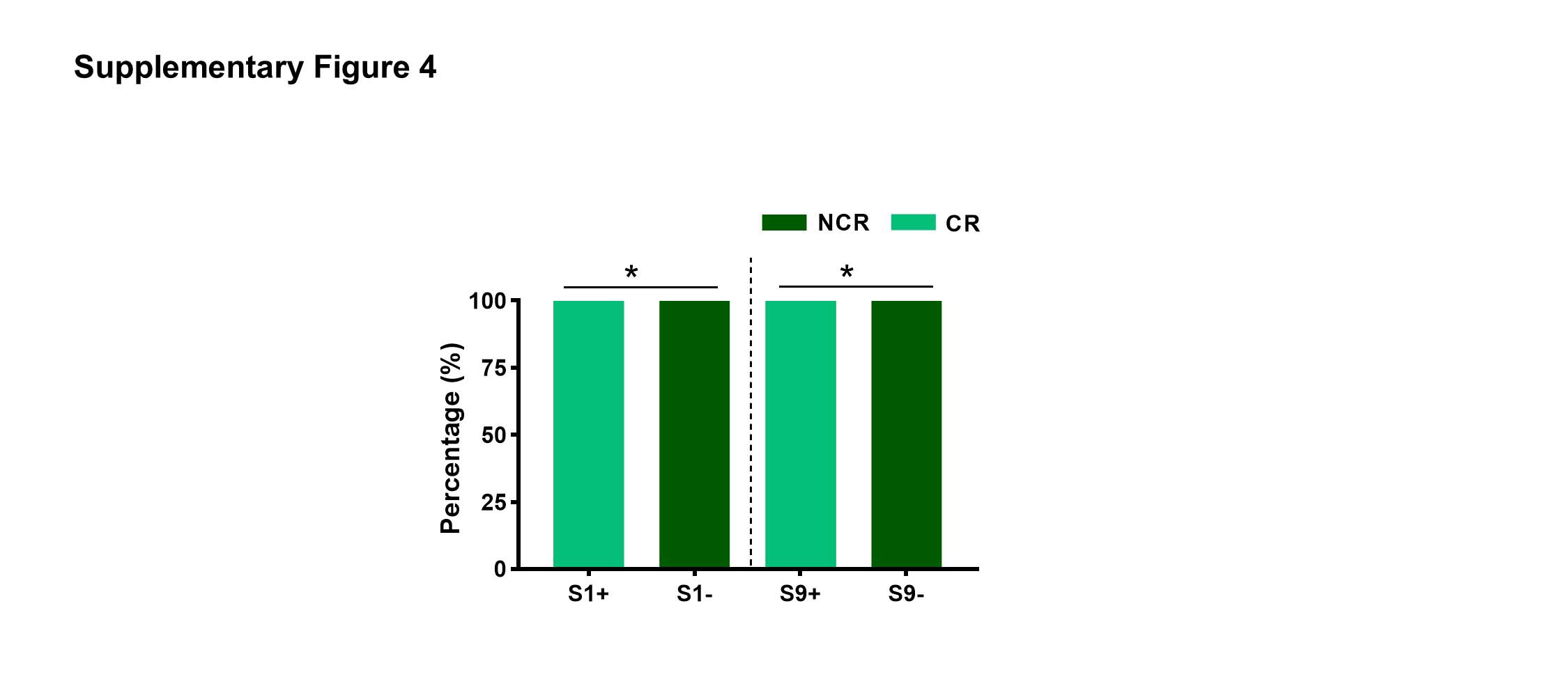

Supplement: Supplementary Figure 4 — The proportion of patients with different treatment responses in the S1+/- or S9+/- groups. Chi-square test. *P < 0.05. [file Image_4.jpeg]
